# The use of technology to address loneliness and social isolation among older adults: the role of social care providers

**DOI:** 10.1186/s12889-023-17386-w

**Published:** 2024-01-06

**Authors:** Elisabeth Grey, Fran Baber, Estelle Corbett, David Ellis, Fiona Gillison, Julie Barnett

**Affiliations:** 1 NIHR Applied Research Collaboration West & Bristol Medical School, University of Bristol, Bristol, UK; 2 Department for Health, University of Bath, Bath, UK; 3 Department of Psychology, University of Bath, Bath, UK; 4 School of Management, University of Bath, Bath, UK

**Keywords:** Loneliness, Social isolation, Social interaction, Digital technology, COVID-19

## Abstract

**Background:**

Addressing loneliness and social isolation among older adults remains a public health priority. The restrictions enforced during the COVID-19 pandemic simultaneously heightened the need for services to overcome social isolation and reduce loneliness among older adults, while also limiting social care providers’ ability to deliver these. The aim of this study was to explore the experiences of social care providers in using technology to address loneliness and social isolation among older adults during the pandemic.

**Methods:**

This was a mixed methods study involving an online survey and interviews with providers of older adult social care in Wales, UK. Invitations to participate were sent to commissioners and providers of adult social care services, including those working in the voluntary and community sectors, across all local authorities in Wales. Data was collected between September 2021 and January 2022.

**Results:**

Sixty-one service providers completed the survey, 19 of whom also took part in an interview. Addressing loneliness and isolation among older adults was reported as a key concern by nearly all survey respondents. While telephone calls were the most common means of facilitating social interaction, many service providers also tried to support older adults to make more use of devices that they already had (e.g., smartphones to hold video calls). Where funding was available, organisations purchased devices, such as tablets and smart speakers, for older adults. Analysis of interviews resulted in three themes: (1) The potential and limitations of technology; (2) Individuals’ capabilities, confidence, motivations and values; and (3) The wider system.

**Conclusions:**

Technology was employed in a variety of ways during the pandemic to address loneliness and social isolation among older adults; many service providers continue to use technology in a hybrid system of care now that pandemic-related social restrictions have been lifted. Our findings emphasise a need for technology-assisted interventions to be designed and deployed in alignment with service users’ values, motivations and capabilities. Further, there is a need to better support service providers to assess loneliness and social isolation among older adults, and to acknowledge the important role providers play in helping older adults to adopt technology-assisted interventions.

**Supplementary Information:**

The online version contains supplementary material available at 10.1186/s12889-023-17386-w.

## Background

Loneliness and social isolation pose considerable risk to the health of older adults. They are separate but moderately correlated concepts [1]; social isolation is the objective condition of a lack of social interaction, whereas loneliness is a subjective state in which one’s social needs are perceived not to be met [2]. Both loneliness and social isolation have been associated with increased likelihood of cardiovascular disorders [3], cognitive impairment [4], dementia [5], depression and anxiety [6]. Among older people with chronic disorders, loneliness and isolation are also associated with poor treatment adherence [7], compounding their effects on health outcomes. Although loneliness and social isolation can affect people of all ages [8, 9] and it is important to acknowledge that people can gain social connections in later life (e.g., through new commitments connected to caring for grandchildren) [10], several factors associated with older age can increase an individual’s risk of loneliness [1, 10–13]. This includes an increased likelihood of developing a chronic health condition which, in turn, can prevent an individual from accessing opportunities for social interaction [11, 12]. Physical disability related to chronic disorders and age-related decline can also make it hard for older adults to join social gatherings, increasing their social isolation [13]. In addition, individuals’ social networks can diminish in older age as they retire from employment, relocate, take on caring responsibilities for partners and with the deaths of their peers [1]. At the societal level, socioeconomic inequality is also directly and indirectly associated with loneliness and social isolation among older adults [14, 15], such that those living in the most deprived regions are more likely to experience social exclusion and loneliness.

The prevalence of loneliness and social isolation, particularly among older adults, has the potential to increase rapidly in the next 15 years with a predicted rise of over a third in the number of people living with major illness [16]. While advances in health care mean that more people can remain living at home despite serious illness, their ability to work and socialise in-person will be diminished. In recognition of the negative impact on health and associated cost to society [17], addressing loneliness and social isolation among older adults has been highlighted as an international public health priority by the United Nations and World Health Organization [18]. In the UK, the government published a strategy for improving social connection in 2018 [19], with subsequent annual reports of progress showing that action has mainly taken place in third sector organisations and local authority health and social care teams [20, 21]. With restraints on funding for these sectors, there is often a reliance on volunteers to deliver interventions aimed at addressing loneliness and social isolation; while volunteer services have been shown to benefit both lonely/isolated service users and volunteers [22], they are challenging to evaluate and difficult to sustain long-term [23].

Technology interventions, particularly those involving digital and Internet-based technology, may have potential for helping to address loneliness and social isolation among older adults. Communication technologies specifically can overcome physical barriers that prevent people meeting in person and facilitate social connection. Over the past two decades, numerous technology-assisted interventions for older adults have been tested. However, evidence of their effect on loneliness and social isolation is mixed, limited by low quality studies and reporting [24, 25]. The strongest evidence so far exists for digital interventions that directly enable older adults to interact with friends, family and the community (e.g., helping individuals to use videoconferencing software) [24, 25]. General training in how to use digital technology for communication also shows promise for reducing loneliness among older adults [25] and more research is needed on the effectiveness of other technology interventions, such as virtual assistants, robotics and digitally delivered group activities.

Various factors determining older adults’ adoption of social technologies have been identified in the literature and included within theoretical models of technology use (e.g., 26, 27). These factors have been categorised by Barbosa Neves and colleagues [26] as falling into three groups: attitudinal - relating to the individual’s interest in the technology, confidence, expectations etc.; functional - relating to access and usability of the technology as well as the individual’s digital skills; and physical - relating to the individual’s physical capabilities and limitations in using technologies. However, relatively little research has considered how these factors interact with socio-contextual influences [26]. For example, family, friends and intervention providers can act to bolster or diminish an older person’s confidence, in turn affecting their use of a technology [27]. Despite this, technology-assisted interventions have tended to be designed with only the older adult end-user in mind, neglecting the role of service providers, friends and family, which can limit their ability to help older adults adopt the interventions [28]. A better understanding of how the social environment relates to older adults’ uptake of technology, including identifying important social actors and their capabilities and needs, could help us to design and deploy interventions that are more appropriate to an individual’s circumstances and thus likely to be effective.

Prior to the COVID-19 pandemic, efforts to address loneliness and social isolation among older adults had mainly focused on increasing opportunities for in-person interactions [29]. The social restrictions imposed to curb the spread of COVID-19, however, meant that organisations working with older adults had to change how they delivered services [30, 31]. Simultaneously, the restrictions raised the profile of social isolation and loneliness as areas of concern, increasing the need for interventions to facilitate social connections. As much of society moved online for business and social purposes with the onset of the pandemic, the relatively lower levels of engagement with technology among older adults [32] became more problematic. Thus, when technology interventions were the main tools available to address loneliness and social isolation among older adults, service providers first had to contend with the digital exclusion of their service users. As digital technology has become more pervasive in society, and indeed the use of technology is now necessary to access essential services such as health care and banking, supporting older adults to adopt technology has become a priority [26]. Added to this, the pandemic and increasing rates of poverty among older adults in the UK [33] have exacerbated loneliness and social isolation for many, leading to a renewed call for action on building social connections [34].

The aim of the present study was to explore how social care service providers for community-dwelling older adults in Wales used technology to address loneliness and social isolation during the pandemic, what facilitators and challenges they encountered, and their perceptions of the impact of using technology. This study formed part of a larger body of research, commissioned by the Welsh Government and supported by the Wales Centre for Public Policy (WCPP), to identify what could be learned from responses to the COVID-19 pandemic in relation to tackling loneliness and social isolation among older adults. In addition to the survey and interviews conducted with service providers for the present study, the wider project also involved interviews with community-dwelling older adults; findings from these are presented in the project report [35].

## Methods

This mixed methods study took place in two consecutive phases: first, we ran an online survey for service providers; second, we conducted interviews with service providers via telephone or video call. The research team brought a range of experience to the design, conduct and analysis of this study, including research expertise in older adults’ health and wellbeing, social connection and loneliness, the integration of digital technology in society, and the development and evaluation of digital interventions. None of the researchers were older adults and we did not have direct experience of social and community services in Wales. Data collection for the survey took place between September and November 2021; interviews were conducted in December 2021 and January 2022 – during this period, the UK Government enforced compulsory use of face masks in public indoor venues and encouraged working from home where possible (from 10th December to 27th January) to prevent the spread of the Omicron variant of COVID-19.

### Participants and recruitment

Adult social care commissioners and providers in Wales, including those working in the voluntary and community sectors, were invited to take part in the study. Invitations were distributed to individuals and organisations known to be commissioning or delivering community-based services to older adults (aged 65 years and over); recruitment was supported by the commissioners of this research, who helped distribute invitations. Recipients were requested to forward the invitation to colleagues also working in this field. This recruitment strategy precludes calculating a response rate as we did not track the number of people to whom gatekeepers forwarded the survey invitation. To encourage participation, three reminders were sent and entry to a prize draw to win one of three shopping vouchers (£50, £30 and £20) was offered as an incentive. Email invites stated that the researchers were interested in understanding if and how technology was being used to build social connections and tackle loneliness and social isolation among older adults, but they did not specify that respondents needed to have experience of this. In the survey, respondents were also asked to indicate if they would be interested in taking part in an interview; from those who were, purposive sampling was employed to maximise variation in terms of the extent to which technology was used to support service users, geographical location, job roles, age and gender. Recruitment to interviews ended when the research team considered the dataset to contain sufficient variety and depth of information on providers’ experiences to enable in-depth analysis and comparisons.

### Data collection

The online survey contained a mix of closed and open-ended questions to ascertain: background information on respondents and their organisations; the types of and extent to which technology had been used; the extent to which addressing loneliness and social isolation was an organisational priority; barriers and facilitators to using technology during the pandemic; challenges and benefits from using technology; and plans for future use of technology to address loneliness and isolation among service users. A copy of the survey is available in Supplementary file 1. The survey was available in English and Welsh language versions; free-text responses in Welsh were translated to English prior to analysis.

Interviews were semi-structured and covered: the nature of organisations’ focus on loneliness and social isolation; the role of technology to address this in the pandemic; who the technology worked best for, how and why; positive and negative impacts of using technology to address loneliness and social isolation; and future plans for technology use as pandemic restrictions were lifted. The researchers conducting the interviews could access and refer to the interviewees’ survey responses to prompt further discussion. The interview schedule was developed by the research team with input from an experts-by-experience group of older adults and the project steering group (consisting of colleagues in the WCPP and Welsh Government); a copy can be found in Supplementary file 2. Oral consent was taken at the start of calls before the interviews began. Anonymised transcripts have been deposited in the University of Bath data archive and are available for research [36].

Most interviews were conducted by EC and FB, both research assistants with masters’ degrees in Psychology and experienced in conducting remote interviews. One interview was conducted in Welsh by a researcher in the WCPP. All interviews were transcribed verbatim (and translated into English if necessary) for analysis.

### Analysis

Descriptive statistical analysis of quantitative survey data was conducted using Microsoft Excel and SPSS software. Content analysis of free-text survey responses was conducted to identify and summarise recurring and contrasting issues.

Interview transcripts were analysed using the ‘codebook’ variant of thematic analysis [37], taking a structured approach to coding where themes are considered as domain summaries. Data coding was conducted by EC and FB using NVivo version 12 software. The researchers first read through the transcripts and their field notes, then drew up a coding framework in discussion with EG and JB; the framework developed iteratively as the analysis progressed. An inductive approach was taken to generating codes and themes relevant to the research aims, aiming to represent the range of views among participants, highlighting commonalities and differences in experiences. We approached the analysis from a critical realist stance [38], believing that reality is understood through individuals’ perceptions and interpretations, as reported in their responses to interview and survey questions. Organisation of codes into the themes presented here was conducted by EG and reviewed and agreed by all authors. Findings from the analyses of both phases were reviewed by all authors.

## Results

In this section, we first describe the sample, then provide an overview of the use of technology within their organisations, drawing mainly on responses to the survey. Three themes from the analysis are then presented, illustrated by quotations from the interviews as well as survey data.

The survey was accessed by 128 people, 39 of whom did not respond to any questions and a further 28 completed less than 55% of the survey, providing little information on their organisations’ use of technology with service users. The findings reported here focus only on the 61 respondents who completed all sections of the survey. There were no statistically significant differences between those who completed the whole survey and those who only partially completed it in terms of how often they used devices to communicate with service users, or the extent to which preventing or reducing loneliness and social isolation was a priority of their role or their organisation.

The survey sample included public, private and third sector organisations providing services in all 22 local authorities of Wales, many providing services in multiple authorities. Respondents ranged in age group from 18 to 24 (3%) to over 64 (13%) with most being 45–54 (30%); forty-eight respondents (79%) were female. The majority of respondents (61%) had over 10 years’ experience working in the care, voluntary or community sector and all but seven (11%) had direct contact with service users. Two respondents were commissioners of adult social care, the rest worked or volunteered for organisations providing adult social care or support for older adults. Respondents’ organisations predominantly served vulnerable or marginalised populations, with only four (7%) being open to all; 29 respondent organisations (predominantly or exclusively) supported adults with physical disability or frailty; 19 supported adults with mental or cognitive health disorders; 10 supported adults with learning disabilities or low education; 13 supported a majority of older adults on low incomes.

Thirty-six survey respondents indicated they were interested in participating in an interview; of these, 28 were contacted but nine did not respond. In total, 19 interviews with service providers were conducted, lasting between 30 and 62 min (mean length = 43 min). Interviewees represented a range of public, private and voluntary sector organisations; further descriptive information is provided in Table 1.

**Table 1:** Characteristics of interview participants

Participant Number	Gender	Age	Role	Length of service in the care, voluntary or community sector
P001	Female	45–54	Case worker	5–10 years
P002	Male	55–64	Development officer	5–10 years
P003	Male	55–64	Co-ordinator	> 20 years
P004	Male	25–34	Assistant into Work Manager	10–15 years
P005	Female	55–64	Programme manager	15–20 years
P006	Female	55–64	Social worker	> 20 years
P007	Female	45–54	Outreach/information officer	2–5 years
P008	Female	45–54	Project manager	10–15 years
P009	Female	64+	Appointed volunteer	> 20 years
P010	Female	64+	Co-ordinator	> 20 years
P011	Female	35–44	Principal officer	> 20 years
P012	Female	35–44	Responsible individual	2–5 years
P013	Female	45–54	Managing director	> 20 years
P014	Male	55–64	Co-ordinator	5–10 years
P015	Female	45–54	Manager	15–20 years
P016	Female	25–34	Co-ordinator	2–5 years
P017	Female	55–64	Community agent	> 20 years
P018	Female	55–64	Community agent	2–5 years
P019	Female	45–54	Engagement lead	> 20 years

### Organisational context

Survey respondents were asked about device and software use within their organisations (i.e., with colleagues). All but one respondent reported frequently using desktops, laptops and/or tablets, and all respondents used either landlines, mobiles or smartphones to communicate in their organisations, the most popular device being a smartphone (used by 82% of respondents). Email, video calling, instant messaging (SMS or WhatsApp) and social media posting were all frequently used by the majority of respondents’ organisations. This suggests that all service providers had at least some experience with digital technology.

The most commonly used technology to communicate with older adult service users were telephones, with all but one respondent reporting frequent use of landlines, mobiles or smartphones. Frequent use of email, video calls and instant messaging with older adult service users were reported by just under half of respondents (48%, 43% and 44%, respectively). This aligns with interviewees’ perceptions that their service users had a preference for telephone calls due to their familiarity and ease.

**Fig. 1:**
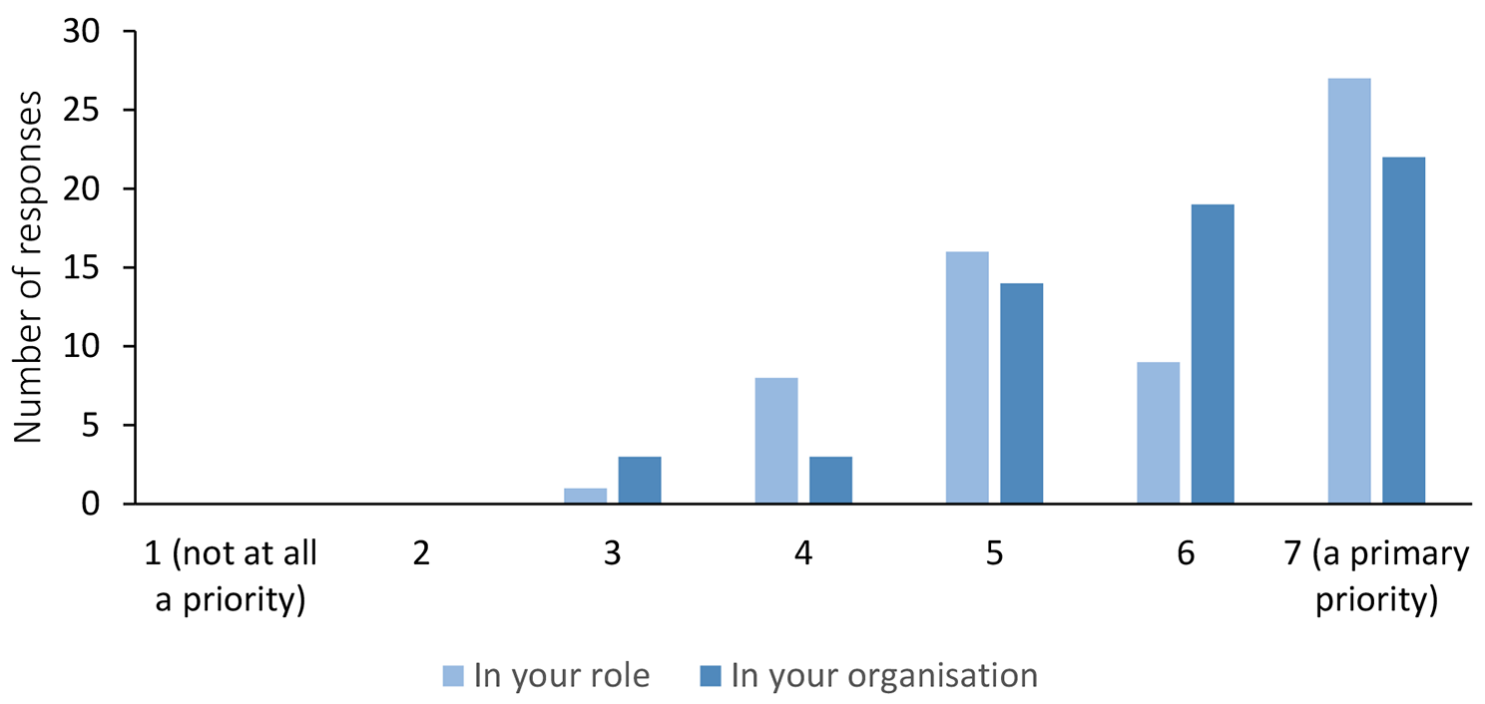
Extent to which preventing/reducing service user loneliness and social isolation was a priority during COVID-19

Preventing or reducing service user loneliness and social isolation during the pandemic was a priority for the majority of respondents and their organisations (Fig. 1). However, 38% of respondents stated that their organisations did not assess or measure loneliness in their service users, and among those that did, assessment methods ranged from using formal measures (e.g., routinely collected induction questionnaires) and structured conversations, through auditing of support requests received from service users, to informal questioning by staff.

As well as contacting service users directly, mostly by telephone, to prevent or reduce loneliness and social isolation during the pandemic, almost all survey respondents (93%) stated that their organisations had encouraged or enabled service users to communicate with friends, family, support groups or other service users. About half of respondents (54%) specified this was done through the provision of technology or technology support, including loaning of tablets, installation of smart home devices and assistive technology (e.g., Amazon Alexa, Komp), and training sessions on how to use video calling and social media platforms.

**Fig. 2:**
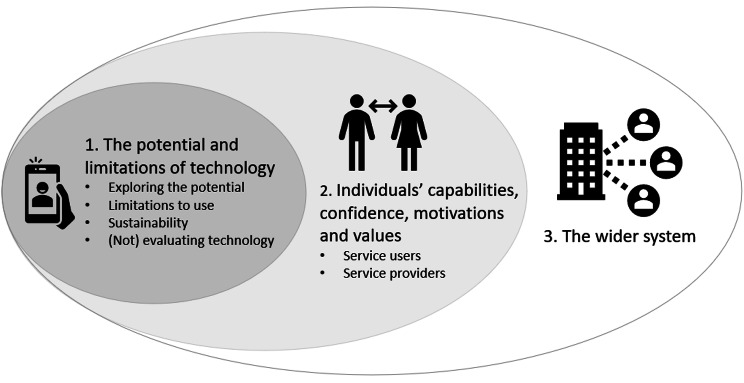
Organisation of themes developed from the analysis of interviews with service providers

The three themes and their sub-themes, developed from the qualitative analysis, are displayed in Fig. 2.

## Theme 1: the potential and limitations of technology

This theme encompasses service providers’ experiences of the variability in appropriateness of different devices and their functions for different purposes.

### Exploring the potential

As highlighted above, telephones – whether traditional landline devices or smartphones – played a central role in organisations’ efforts to prevent and reduce loneliness and social isolation. When social restrictions were first enforced during the pandemic, phone calls were seen as the most accessible means of continuing some level of support as all service users had phones and were familiar with making calls. However, the limitations of traditional, audio-only calls spurred on some service providers and users to try video call platforms such as Zoom and FaceTime.

*Phones are a big necessity for all of us but you can’t see people’s reactions and they can’t see you, you’re just a voice. It’s limiting, essential but limited. You can’t see somebody that’s suffering from depression on the other end of the phone unless they physically talk about it, which often they don’t but when you’re face to face with somebody, you see depression in people’s eyes, it’s a whole different thing… Now we use technology, Zoom calls, Skype sometimes, it’s still limited to those who want to do it and can do it (P009).*

*Although people can still phone people, it wasn’t the same. It was that they wanted to see each other and especially the grandmothers and grandfathers who had great grandchildren or young grandchildren. It was very important for them to be able to either Facetime or video call or be able to share photos and videos with each other by email (P016).*

The majority of survey respondents (67%) found that older adults started using applications on their devices during the pandemic that they had not used before in order to connect with others. For example, several service providers received requests from users to help them join WhatsApp or Facebook using smartphones that they had previously only used for phone calls.

*They’re basically wanting to know how to use their mobile phones! How they can get better use out of it (P010).*

*Quite a lot of people wanted things like WhatsApp, so the video conferencing options to be able to do what they needed to do with family (P015).*

Similarly, some interviewees spoke about how their organisations, at the onset of the pandemic, had tried to make better use of technology to support service users’ independence when they were unable to provide in-person support. For example, some organisations were able to set up smart speakers in service users’ homes so that they could use the in-built virtual assistant technology (e.g., Amazon Alexa) to set reminders or control household tasks they otherwise struggled with due to limited mobility or memory impairments.

*We were also looking at how we can utilise Alexas… So we have bought Alexas and we’re establishing our Alexa suite so people can come, staff members now will be trained up on exactly what Alexa can support with and how it can link to your heating, how it can link to your doorbells… The possibilities are huge. (P011)*

*He likes the technology with the Alexa because it gives him the freedom to be able to open up his curtains on his own. He doesn’t have to wait for the carers to come in. He doesn’t have to have an extra call. Because his wife, her mobility is pretty poor. So we’ve looked at the technology to make sure that they’re still maintaining a normal life (P001).*

The latter participant (P001) acknowledged that improving people’s ability to maintain their independence could potentially increase their isolation by lowering the need for them to engage with others; however, she felt that having a sense of independence was important for the individual’s confidence, which in turn had a “*knock-on effect*” of boosting their sociability. The smart speakers were also intended to enable service users to easily make calls, however, for one person simply speaking to the device was sufficient to prevent feelings of loneliness.

*She was extremely lonely but didn’t want to interact with people because she didn’t like the whole feeling of going out and socialising. The Alexa has brought more for her because she’s been talking to it. She said it was like a lifeline to her. It’s not so much she was talking to other people, it was more that she was able to talk to something (P001).*

### Limitations to use

These examples show the creativity and openness among both service providers and older adults to explore the potential of readily available technology for reducing loneliness and isolation. However, our findings also highlighted variability in the appropriateness and accessibility of technology, with several difficulties and limitations experienced. The majority of survey respondents and interviewees estimated that at least some of their service users did not use smartphones, computers or smart devices. While in some cases this was partly due to the individuals’ attitudes and preferences (discussed in the next theme), factors inherent to the technology also presented barriers, particularly for the high proportion of service users living with physical or cognitive disabilities. For instance, speech difficulties and strong regional accents caused problems for voice recognition software and needing to navigate multiple steps in order to access a particular function was a struggle for people with memory impairment. Touchscreens and small keypads were also difficult for older adults with limited dexterity, although one voluntary sector organisation found that providing tasks and games was an effective way of improving service users’ skills and confidence with handheld devices.

*We gave them tasks to do, things like if you go for a walk, take your tablet with you, walk to the end of the road and take a picture of anything red … Games was a good one to encourage people to use techniques, so word searches encouraged people to learn how to swipe and to tap (P005).*

Data access was also cited as an important barrier by some service providers, particularly those serving people living in rural areas or on low incomes. Some interviewees reported that their organisations were able to purchase data or broadband connections for service users, however, this was only for a limited period. An organisation in one local authority tried to overcome difficulties in the use of technology and data access by providing service users with Komp devices – simplified computers that are operated using one large button, come with built-in 4G and are solely for incoming calls and messages. While the limited functionality of the Komp was a key enabler for some older adults, being seen as more secure, others found it frustrating.

*the younger people don’t particularly like it [the Komp] either because they don’t like the idea of not being able to call out and people calling in. So again it’s that people can come into your home and speak to you when they want, not when you want. So we’ve only used it for the older people (P001).*

As this quotation illustrates, different functions and capabilities of technology will be important for different service users. Therefore, organisations will likely need to be knowledgeable about, if not provide, a variety of different devices and applications in order to meet the individual needs of their service users.

### Sustainability

During the height of the pandemic, individuals and organisations in Wales were able to access grants for technology and data specifically to prevent digital exclusion and loneliness. Many of the interviewees reported accessing this funding or signposting it to their service users and although there were many examples of its beneficial impacts, questions were raised over the long-term sustainability of such schemes. Data and device provision by organisations was only for limited periods and service providers pointed out that, for many older adults, the upfront cost of devices and ongoing costs of broadband connections were unaffordable after the subsidised period. In some cases, providers were able to work with other organisations to access further funding but this varied regionally and others reported that some of their service users returned the devices.

*We’ve got this loan scheme and we’re supporting individuals for a period of time, for no longer than three months, then the local asset coordinators then will support that individual to either purchase a tablet themselves or find another grant or whatever, and then that tablet could go on to another individual. (P003)*

*They couldn’t really afford to be paying £15 for a SIM card when they’re on pension credits. Another community council actually paid for the SIM cards but mine wouldn’t… Some people like that lady stopped because they couldn’t master the tablet and they couldn’t afford the SIM card so cost came into it (P017).*

Another aspect of sustainability raised by the service providers was the resource intensive nature of providing technical support to older adults and their families. This was an ongoing role and required organisations to keep up with advances in technology in order to best advise their service users. Providing devices to older adults who had little experience of digital technology necessitated a lengthy set-up process and, if the individual experienced any problems with the device, they tended to expect the provider to resolve these. Some organisations recruited volunteers or were able to employ a member of staff to focus on providing technical support, but some organisations could not and found they were unable to meet service users’ needs.

*The only way - and it is difficult - is personal supervision, one-to-one… it’s been very difficult but if we want older people to really embrace this, we need to be aware that we have to hold them by the hand … you can’t just give them the stuff and say, “there you are, I’ve shown you how to logon, I’ve shown you how to open it up, it’s all yours”, that’s not enough (P009).*

*We couldn’t spend lots of time sitting with them and telling them how to use it … So we’ve linked quite a lot with our third sector groups locally… digital buddies who’ve been able to try and do some phone conversations (P015).*

*In terms of purchasing and setting things up, if there’s no family [to help], it’s like “Okay, well, have a computer”. That would need setting up and that would need somebody from some organisation that would have to do that (P006).*

Using adapted devices, which service providers could monitor remotely, helped overcome some of the challenges in providing technical support. These devices enabled quick identification of problems, although it should be noted that service providers reported that a concern among some older adults was the idea of digital surveillance, feeling it intruded on their privacy. Monitoring device use therefore needed to be negotiated with individual service users and their families.

### (Not) evaluating technology

A final point to note within this theme is that, similar to assessment of older adults’ loneliness and isolation, few organisations were evaluating any technology-assisted intervention they had implemented, thus limiting their ability to recognise the potential and limitations of the technology. The evaluations that were conducted consisted of gathering feedback from service users, but this did not seem to be done routinely or using robust measures. Reasons cited for little or no evaluation included lack of time and resources, and low response rates from older adults to previous evaluation surveys. Despite this, service providers were aware that robust evaluation could help them demonstrate impact to acquire further funding, as well as indicate how best to allocate resources for future service provision.

*I’d like something more formal. I’d like something like an external evaluation of it… a monitor around it and evaluate where we’ve been putting the money, rather than it just being a scattergun, which is easy to do, but we don’t know if it’s making any difference and we want to be able to prove it (P011).*

## Theme 2: individuals’ capabilities, confidence, motivations and values

As well as factors inherent to the technology, our analysis highlighted the importance of individual service users’ and providers’ abilities and attitudes in the adoption and use of technology to prevent loneliness.

### Service users

A commonly reported enabler for technology use among older adults was having a specific goal that a device or application would help them achieve; that is, mastering technology did not seem to be a sufficient motivation in itself but rather a means to a desired end. Seeing family and friends was a key driver for service users to try new devices and applications, but attending group activities (such as singing or exercise classes) and accessing services (e.g., ordering repeat prescriptions) were also motivators. One interviewee illustrated this goal-directed adoption of technology in the case of a woman who had sought their help with her tablet and home Wi-Fi after trying to make use of public Wi-Fi in order to join her online church group:

*Every Sunday there was the church that she used to attend that went online, but she used to come to the library, not open on a Sunday, and sit outside, just so she could connect to the Wi-Fi. She had Wi-Fi in her home, she didn’t know how to connect to it or anything like that, and there was no real support network… it’s just a story that sticks in my mind because I think of this old lady sat outside the hub on a rainy day [connecting] with her church group, which is the kind of thing that we were trying to avoid (P004).*

While the service user’s values and specific outcomes they wished to achieve often determined the type of device or application that they initially used (e.g., WhatsApp or Facetime to connect with family), the increased familiarity with technology in some cases helped to build individuals’ confidence and lead them to independently explore other applications.

*I think probably the positive effects are that people feel empowered to go and use [Zoom] for other reasons as well, they may think “I can do this for [singing group] so what other groups are there that I can log onto and use? What other services are there?”. The potential is endless really, a lot of people I know have book clubs, reading clubs and things like that (P008).*

Importantly, for such independent use to occur, service users needed to feel confident in their abilities. This varied on an individual basis: while some started with basic skills and were willing to try again after making mistakes, others were more fearful and required more support and encouragement to overcome initial difficulties.

*Those that already could use a computer but only used it perhaps to tinker about, order something online, once they’d got into Zoom they progressed very quickly to really enjoying that process and their wellbeing improved and they gained confidence. To gain confidence, you improve your feeling of wellbeing and your self-worth goes up 100-fold, which is wonderful (P009).*

*I think people weren’t sure how to use the technology and it was just trying to overcome that and almost hand-holding through the process of joining and getting people to join, which took a lot more work than we thought it would actually (P007).*

Service providers encountered various reasons for older adults’ fear of using technology, including concern that they would inadvertently break expensive devices or appear foolish in online group activities if they *“mess up”* by not navigating the platform correctly. Surveillance and other people being able to access personal details were also off-putting. However, the chief source of fear was online scams – a fear that, in some cases, was shared by service users’ family members.

*It’s the fact that somebody can access their bank accounts and their hard-earned pensions. And so there is a fear. It’s a bit like having smart meters as well. There’s a lot of resistance against having smart stuff (P002).*

*Surprisingly enough, it was family members that were not very encouraging. We came across a lot of family members who didn’t want their parents or grandparents to perhaps have access to technology… they were frightened that they might be scammed because they wouldn’t be aware of what could happen (P005)*

One provider tried to allay these fears by providing managed devices with scam and malware blocking technology installed. Others offered training and education on how to use different devices and online services, and avoid scams, as well as reassurance that technology can be replaced or mended. This could be a time-consuming process for service providers, particularly as many felt this was best done in-person and tailored to individual needs, but it was seen as important for helping older adults to get past the first hurdle in using technology and on the path to independent, confident use. However, for some service users even one-to-one sessions with a provider were not enough to overcome their concerns.

*Having the managed system I think offers a level of protection that people are comfortable with, and certainly family members are comfortable with as well, knowing that mum and dad are not going to be able to enter their bank details and have all their life savings taken from them (P015).*

*when we were introducing things like [tablets] there was that hesitancy, there was that little bit of fear and I think some people will never get over that until the time we can go and sit there and drop it on the floor and pick it up and go, “Look, it still works.” I suppose it depends on the technology that we’re talking about. Some of it, I think we can get over that fear by simply saying, “If it breaks social services will give you a new one,” as opposed to, “If it breaks you have to pay £200.” (P013)*.

As suggested in the last quotation, encouragement, education and support by service providers will not be enough to get all older adults digitally engaged. Service providers recognised that in-person and non-digital means of preventing loneliness would always need to be provided for older adults who were unable to learn how to use new technologies (e.g., due to certain cognitive impairments) or were simply not interested in doing so. However, as one interviewee highlighted, lack of interest could indicate underlying poor mental health, perhaps stemming from social isolation and loneliness; in such cases, more intensive, holistic support may be required, with technology being introduced at a later stage.

*Sometimes if somebody has been on their own for so long and they’re like “Oh I’m not interested”. But there’s a lot of things that become apparent that perhaps that social isolation, that loneliness is impacting on their health and wellbeing. And there’s a piece of work there that needs to be done in order to support that person to a point where they may feel that “actually do you know what, I do want to connect with people” (P006).*

### Service providers

Survey and interview responses illustrated that there was not only diversity in the skills and attitudes of service users regarding technology, but also service providers. We asked survey respondents the extent to which they felt the use of technology to reduce loneliness and social isolation among their service users was appropriate and beneficial. Forty-four people (72%) felt it was appropriate, citing the benefits they had witnessed as the reason for this, while also acknowledging the importance of maintaining in-person contact. Two people felt technology was inappropriate for their service users and a further 23% were ambivalent; these respondents explained that not all of their service users were able or willing to use technology, suggesting that technology should be seen as an *‘additional tool’* but not a replacement for face-to-face contact. All respondents answered that technology had been at least ‘slightly beneficial’ in helping them to reduce loneliness and social isolation. Again, those who gave less positive ratings (i.e., slightly or moderately (28%) rather than very or extremely beneficial) highlighted the potential to exclude service users who lacked confidence, motivation or access. Those who felt technology had been more beneficial provided examples of new social connections that their service users had made and cited positive feedback they had received. These findings suggest that the differences in attitudes towards technology may be reflective of the different support that these respondents were able to provide as well as the different populations they served. That is, those who felt more able to meet their service users’ needs with the technology they had available, may hold more positive views towards it.

A further influence on service providers’ attitudes towards technology is likely to come from their confidence in using it. While all interviewees were comfortable using email and web browsing, several admitted that, at least at the start of the pandemic, they were not confident using video calling platforms and shared older adults’ fears about scams. In order to support their service users, these providers quickly had to learn how to use new software and devices – but this was approached more enthusiastically by some compared to others.

*I have to say, even at my age, I’ll text my children or anybody and keep in touch, give them a call, but I don’t think to video call them. I don’t think to, because yes, we use Teams now, and that’s more and more, but very often in work I will e-mail as opposed to video call, when a quick video call would suffice and save time. (P014)*

*I mean I knew nothing about Zoom. I don’t think any of us did really before the pandemic. Everybody always says to me, “Oh, you’re an expert on it.” I was like, “Well, just trial and error, isn’t it? You just learn as you go along” (P104)*.

A few organisations acknowledged the need to train and support their staff to build familiarity and confidence with new technology, providing devices to try and seeking training courses that their staff could complete. Other service providers had to learn and improve their skills in their own time.

*The volunteers, some of them weren’t very confident… I think they were saying “what if I show them how to do something and they got it wrong, or what if I didn’t know something?” We put some training on. We worked closely with [company] and did a six week training course training people how to be digital companions and how to support people. And a lot of that was about confidence and confidence building (P007)*.

*We had to try all of these tech at first. So that was really good, and sometimes it was quite funny. We’d be in meetings and then we’d mention Alexa and then all of our Alexas would go off… So for us we learnt stuff from it as well. We were learning about all the different, you know, the opportunities (P011).*

Having mastered new technology, the service providers then had to teach others how to use it, which, as several pointed out, was a separate skill that again took time to learn.

*Using technology yourself is one thing… Then trying to support somebody and talk someone through who has got very little digital skill is quite difficult. Trying to explain “look to the bottom left hand side of your screen, it’s the thing that looks like an ice cream”. Just trying to put it into layman’s terms. It really makes you think, and it’s a different skill to be able to show somebody else what to do to be able to do it yourself (P007).*

These responses indicate a need for organisations to assess not only their service users’ confidence, skills and potential motivators for using technology but also those of their staff and volunteers. Understanding these factors can enable appropriate support and resources to be provided for both groups; this should lead to more positive experiences, which in turn may make service providers and users more receptive to technology for preventing loneliness [39]. This will be important for future service provision as most survey respondents (87%) reported that at least some of their support would continue to be provided remotely.

## Theme 3: the wider system

Service providers were aware that they could have only limited impact on older adults’ loneliness and social isolation, and that, for lasting impact, support would also need to come from the wider community. Both survey and interview respondents reported that service users’ families were often enlisted to provide additional help in setting up devices and showing older adults how to use different applications. However, not all service users had family nearby and, if they did, their relatives were sometimes older adults themselves with no greater confidence or skills in using technology. While some organisations were able to offer support and training for relatives as well as the service users, this was beyond the capacity of some.

*I do tend to talk to the daughter or the son, and that’s been quite useful, because often they are IT literate and I can send them info, and they can then pass it on. So that’s been quite useful and I would say I’ve spoken to a lot more relatives since the pandemic than I did before (P018).*

*Being an older group of people their families, including sons and daughters, they’re pretty old and definitely husbands and wives are old and a lot of them, maybe even the majority of them, when we were saying, “Can you send us your Skype user ID?” Had no idea, had never done things like that (P012).*

Recognising that they could not meet the needs of service users on their own, organisations also sought support from other providers and groups in the community. This seemed to rely on the organisations having existing connections and networks in place, and required service providers to take on an additional task of planning and coordinating work across organisations.

*We did have one group that, pre-COVID, basically had the local year six children coming in to help the older guys with their tablets (P002).*

*We’ve had some really good support from the council… Because we are place based, I’ve got connections within the community to have people go along and give them a hand to get the computer up and running, or things up and running. That’s something we consciously look to support with solutions for (P014).*

Service providers were also aware that they were not reaching certain groups of older adults who could benefit from their support. There was a tension between supporting people to adopt technology so that services could move online, while knowing that a significant proportion would or could not use technology and so the digital divide would increase. Service providers highlighted that the people they were unable to reach tended to be those in already marginalised groups, including those with learning and cognitive disabilities, people whose first language is not English (or Welsh) and those living in very rural locations.

*If somebody has dementia and perhaps they lack capacity to understand and even consent perhaps to have some form of technology which would link them to other people and reduce their social isolation for example. At the end of the day you can only give people information and advice about things (P006).*

*Most of our sessions have been for people who either speak Welsh or who speak English. We, even now, don’t have the resources to expand to groups who don’t speak Welsh or don’t speak English. So we haven’t been able to give as much… Well, we haven’t been able to give any focus to them, unfortunately (P019).*

*There were still those people for whom being excluded was just the norm. I’ve said before, there are areas of North Wales where you can’t get a mobile signal. There are areas of North Wales where the internet connection is rubbish (P013).*

## Discussion

This study has explored social care service providers’ experiences of using technology to reduce loneliness and social isolation among older adults in Wales during the first two years of the COVID-19 pandemic. We found that addressing loneliness and social isolation was a key concern for organisations and they sought to make use of the technology that their service users already owned (landlines and smartphones), as well as procuring new devices (e.g., smart speakers, tablets), to help people stay connected with friends, family and the community. In order to do this, many service providers rapidly had to acquire new skills, find and apply for funding, and develop new ways of working in coordination with other organisations. It is notable, however, that very few organisations formally measured or assessed loneliness and social isolation among their service users, or conducted evaluations of the technology-assisted interventions they implemented. Without gathering robust data on loneliness and social isolation, organisations may be unable to provide evidence that resources need to be allocated to address these issues. On the other hand, organisations may implement interventions aimed at reducing loneliness and social isolation but, without taking baseline and follow-up measures, will be unable to assess whether or not the interventions are effective.

In line with previous research [27, 40, 41], our participants reported diversity in older adults’ confidence, motivation, skills, abilities and access with regards to technology. These factors also map on to theoretical constructs thought to determine individuals’ technology adoption [42, 43] – categorised by Barbosa Neves and colleagues as attitudinal, functional and physical [26] - indicating a need to align interventions with service users’ individual motivations, capabilities and circumstances in order for them to be adopted [27, 44, 45]. For example, an evidence review of the use of video call technology to improve social connectedness highlighted that, to enhance uptake, the design of software and devices needs to be informed by older adults’ competency and conditions [46]. Research has shown that stereotypes of older adults as technophobic, incapable or unwilling to engage with technology are often inaccurate [47] and it is important for service providers to assess each older adult’s needs individually. To facilitate this, service providers could conduct brief assessments of service users’ technological readiness at their initial engagement with the service. Assessments could be based on technology use theory to include questions on confidence, motivation and values with regards to social connection and technology. This could help to identify challenges a person may face in adopting a new technology and also potential ‘pull factors’. For example, if someone places high importance on being part of a local community group, focusing an intervention on helping them do this via digital technology would match the intervention to their values, and thus they may be more motivated to engage. As we found in this study, building service users’ confidence and motivation to try technology is a key barrier to overcome in order to enable further, independent use of technology. However, care must be taken in assessing current circumstances and capabilities as this process can highlight an individual’s lack of social connections or limited knowledge of technology, which may emphasise their loneliness and diminish their self-esteem [28, 48]. Research is therefore needed to develop acceptable and effective means of assessing older adults’ technological readiness and social circumstances that are feasible to incorporate in standard care.

The input of service users’ relatives in such assessments may also be important; we found that relatives can act as both facilitators and barriers to older adults’ adoption of technology. This supports previous research illustrating that relatives often provide encouragement and technical support as well as being the impetus for older adults to use technology (i.e., in order to connect with their relatives) [49–51], though they may also underestimate older adults’ ability or incorrectly assume that they will be accepting of monitored devices [51, 52]. Involving relatives in initial assessments with service users could help identify potential sources of additional support for adopting technology or potential challenges that may require supplementary intervention (e.g., provision of information to relatives on the safety netting software deployed on managed devices). However, involving relatives could, at least initially, increase burden on service providers if they have to support both the service user and their family. Indeed, many participants in our study reported informally involving service users’ relatives in the provision of support, not only to promote engagement with technology but also because their organisation had limited capacity and so needed to enlist others to meet their service users’ needs. Previous work on personalising technology-assisted interventions for older adults has also highlighted the limited time care providers have for gathering background information on people and engaging with their families [53]. Further work may thus be needed to identify feasible ways to include service users’ relatives in their service plans in ways that minimise pressure on service providers.

As people’s needs and abilities are likely to change over time, for example due to deterioration in physical condition or improvement in digital literacy, ideally assessments of service users’ technical skills, values and motivations would be repeated periodically. This would also assist with evaluating and improving services; for example, identifying which personal characteristics are associated with greater or faster improvement in digital skills could help organisations see which interventions may be most appropriate for different users. In addition, regular tracking of service users’ needs and engagement with interventions could highlight unexpected outcomes. Even with baseline assessment of capabilities and values, it will not be possible to anticipate all the possible challenges and enablers that a person will encounter when using technology. While the identification of unintended risks is obviously important from an ethical perspective, identifying unexpected benefits is also important for informing intervention development [54]. The case described by one of our interviewees of an individual’s loneliness being reduced by conversation with a virtual assistant, provided a helpful reminder that researchers and service providers need to keep an open mind with regards to older adults’ adoption of technology [48].

This study has also demonstrated the diversity in social care service providers’ confidence, attitudes and skills in relation to using technology to reduce loneliness and social isolation among older adults, as well as the differences in availability of devices and support that they face. The abilities and needs of service providers have received relatively less research focus despite the important role service providers can play in determining older adults’ engagement with technology-assisted interventions [27, 55]. Some of our participants expressed a lack of confidence in their own knowledge and skills with technology, as well as in the ability to teach service users; others were unsure that technology-assisted interventions were appropriate for their service users. In order to reassure and encourage older adults, it is important that service providers feel confident not only in their own ability but also in the intervention they are trying to employ. A recent study of a virtual reality (VR) intervention for older adult care home residents found that allowing care home staff time to use and experiment with the VR devices themselves was critical to successful adoption and use of the intervention [56]; this not only helped give staff confidence in the intervention but also increased their self-efficacy to support residents with the devices. Providing training and support in the use of technology for enabling social connection among older adults for service providers, and ensuring they have protected time for training [55–57], could help build confidence and skills, as well as inform how and with whom to use different devices or applications [55, 58]. Training may also encourage volunteer service providers, many of whom cite gaining skills as a reason for volunteering [59]. In addition, involving both service users and providers in the design of technology-assisted interventions, or the deployment of off-the-shelf applications, could help to give both groups confidence in their fitness for purpose and ensure that organisational factors, such as time required to introduce a new device, are feasible for service providers [46, 55, 56, 58]. While this was not a priority in the context of a pandemic, when rapid intervention was required, the lifting of social restrictions makes co-production more possible. Indeed the COVID-19 pandemic and increasing prevalence of poverty among older adults in the UK seem to have exacerbated loneliness among many communities [34], emphasising the need for action to increase digital capability among older adults and service providers.

Although digital interventions should not be seen as a replacement for face-to-face socialising, technology can provide complementary functions to enhance traditional social interaction or maintain contact during periods where in-person communication is not possible. Older adults social care service users are particularly vulnerable to experiencing periods where they are unable to access their usual social activities, for example, due to worsening illness or limited money. It is therefore important that the digital skills gained by service users and providers during the pandemic are not lost but are harnessed and enhanced, and further efforts are made to ensure those who could most benefit from digital interventions are enabled to do so. In the UK, a recent Parliament report called for renewed action to tackle digital exclusion, specifically recommending that providers of interventions to reach those who are digitally excluded are enabled to access adequate funding and resources [60]. While many service providers in our study were able to access some form of support, either in the form of funding to purchase devices for their service users or training for their staff, there was no central coordination to assist organisations in finding this support. Initiating a service or advice hub for providers of older adult social care to inform them of the support, training and funding available, and how to access it, would help to reduce provider burden and, potentially, duplication of effort. This in turn could increase their time for supporting those service users for whom technology-assisted interventions are not suitable or not available, such as people living in regions with no internet coverage. Further research is needed to identify how we can best assess loneliness and social isolation among diverse service users, in ways that are sensitive to change associated with technology-assisted interventions. In the meantime, the UK government has recommended the use of a 4-item measure for loneliness in adults and committed to use this in their surveys [61]. Our findings suggest that organisations may also require support and additional funding to adopt this measure in their routine practice. Although the social restrictions imposed during the height of the pandemic have mostly been lifted, prevalence rates of chronic loneliness in the UK have not reduced [62]. Evidence suggests that there changes in people’s social relationships, brought about in COVID-19 lockdowns, are enduring– for example, ‘pruning’ of social networks to concentrate socialising efforts on close friends and family, a loss of local community engagement [63]. For some people, particularly those with physical and mental disabilities and their carers, these social changes seem to have increased loneliness and social isolation [62, 63]. With the predicted rise in people living at home with major illness [16], this emphasises the need to maintain, if not increase, efforts to relieve loneliness and social isolation.

While training and financial assistance could substantially aid organisations in using technology to reduce loneliness among older adults, whole system change will be required in order to make a lasting difference at scale. Many service providers reported the involvement of service users’ family members and, as has been found in other research [27, 45], this could prove crucial to older adults’ successful engagement with an intervention. However, not all older adults will have family who are willing and able to provide support [28] and, even if they do, this can place an additional burden on service providers to assist not only the service user but also their family. Policy change to support affordable internet access, improved connectivity and coverage, and making online services more accessible as recommended in a recent UK Parliament report [60], is to be welcomed – as highlighted by some participants, there are still regions of the UK without internet coverage. However, there is also a need to develop resources, capacity and skills within the wider society to provide the social and technical support that social care organisations do not have the resources to offer and before internet coverage improves. Research on digital interventions for loneliness and social isolation has tended not to report community outcomes (e.g., social support and cohesion, digital literacy inequalities) or process indicators (e.g., feasibility, acceptance, adherence and cost-effectiveness) [25], yet evidence on these factors would help organisations and policy makers decide how best to allocate their limited resources and should be an area for future research focus. Research suggests that third sector interventions for loneliness that are reliant on a volunteer workforce may not be sustainable without additional paid staff to coordinate the work [23]. Considering the wider system of influences on older adults’ loneliness and social isolation is also important [64]. Factors such as transport, physical infrastructure, employment and economic systems will impact on older adults’ ability to make and sustain social connections, whether traditional or digital; policy and interventions that take account of these factors and act to reduce societal inequality [14, 15] may be more likely to bring about sustainable improvement in loneliness and social isolation. Ultimately, to meet the heterogeneous needs of the older adult population, a variety of initiatives across multiple systems will be needed [65].

## Strengths and limitations

This study supplements the substantial body of research on older adults’ experiences of using technology to address loneliness and social isolation by providing valuable insight from the perspective of social care service providers who work with this population. It is noteworthy that many participants in this study worked for organisations that specifically served socially disadvantaged groups (e.g., people on lower incomes and living with disabilities) – these groups tend to be less-well represented in research and yet are known to be at greater risk for loneliness and social isolation [66, 67]. The samples for both the survey and interviews in this study were diverse in terms of job roles, locations and ages of participants, representing a wide range of service providers. The sample size for the survey was too small to allow definitive statistical comparisons, which may have been partly due to the length of the survey (up to 56 questions) causing fatigue and drop-out; however, the data was sufficient to indicate trends and provide a descriptive backdrop for findings from the analysis of the interviews.

## Conclusion

Social care service providers in Wales, UK, employed technology in a variety of ways during the pandemic to address loneliness and social isolation among older adults, and many are continuing to do so now that social restrictions have been lifted. Barriers and facilitators to using technology operated at multiple levels: the device/application; the individual (both service users and providers); the social community (friends and relatives); organisations; and national policy. Our findings support the literature calling for technology-assisted interventions to be designed and deployed in alignment with service users’ values, motivations and capabilities. This research has also highlighted a need to better support service providers to assess loneliness and social isolation among older adults, and to deliver technology-assisted interventions. Connecting organisations across local and regional boundaries, and engaging the support of service users’ friends and family, could help service providers to address loneliness and social isolation among older adults.

### Electronic supplementary material

Below is the link to the electronic supplementary material.

Supplementary Material 1

Supplementary Material 2

## Data Availability

The dataset generated and analysed during the current study is available in the University of Bath Research Data Archive repository, 10.15125/BATH-01346.

## References

[1] Taylor HO (2020). Social isolation’s influence on loneliness among older adults. Clin Soc Work J.

[2] Taylor HO, Cudjoe TKM, Bu F, Lim MH (2023). The state of loneliness and social isolation research: current knowledge and future directions. BMC Public Health.

[3] Valtorta NK, Kanaan M, Gilbody S, Ronzi S, Hanratty B (2016). Loneliness and social isolation as risk factors for coronary Heart Disease and Stroke: systematic review and meta-analysis of longitudinal observational studies. Heart.

[4] Evans IEM, Martyr A, Collins R, Brayne C, Clare L (2019). Social isolation and cognitive function in later life: a systematic review and Meta-analysis. J Alzheimers Dis.

[5] Kuiper JS, Zuidersma M, Oude Voshaar RC, Zuidema SU, van den Heuvel ER, Stolk RP (2015). Social relationships and risk of Dementia: a systematic review and meta-analysis of longitudinal cohort studies. Ageing Res Rev.

[6] Leigh-Hunt N, Bagguley D, Bash K, Turner V, Turnbull S, Valtorta N (2017). An overview of systematic reviews on the public health consequences of social isolation and loneliness. Public Health.

[7] Lu J, Zhang N, Mao D, Wang Y, Wang X (2020). How social isolation and loneliness effect medication adherence among elderly with chronic Diseases: an integrated theory and validated cross-sectional study. Arch Gerontol Geriatr.

[8] Surkalim DL, Luo M, Eres R, Gebel K, van Buskirk J, Bauman A (2022). The prevalence of loneliness across 113 countries: systematic review and meta-analysis. BMJ.

[9] Barreto M, Victor C, Hammond C, Eccles A, Richins MT, Qualter P (2021). Loneliness around the world: age, gender, and cultural differences in loneliness. Pers Indiv Differ.

[10] Dykstra PA, van Tilburg TG, Gierveld JdJ (2005). Changes in older adult loneliness: results from a seven-year longitudinal study. Res Aging.

[11] Barlow MA, Liu SY, Wrosch C (2015). Chronic Illness and loneliness in older adulthood: the role of self-protective control strategies. Health Psychol.

[12] Christiansen J, Lund R, Qualter P, Andersen CM, Pedersen SS, Lasgaard M (2021). Loneliness, social isolation, and Chronic Disease outcomes. Ann Behav Med.

[13] Elder K, Retrum J. Framework for isolation in adults over 50. AARP Foundation. 2012.

[14] Gierveld JJ, TGv T, Dykstra PA, Vangelisti AL, Perlman D (2018). New ways of Theorizing and Conducting Research in the field of loneliness and social isolation. The Cambridge Handbook of Personal relationships.

[15] Victor CR, Pikhartova J (2020). Lonely places or lonely people? Investigating the relationship between loneliness and place of residence. BMC Public Health.

[16] Watt T, Raymond A, Rachet-Jacquet L, Head A, Kypridemos C, Kelly E et al. Health in 2040: projected patterns of illness in England. Retrieved from: https://www.health.org.uk/publications/health-in-2040: The Health Foundation; 2023.

[17] Hawkley LC (2022). Loneliness and health. Nat Reviews Disease Primers.

[18] World Health Organisation. Social isolation and loneliness. Accessed from: https://www.who.int/teams/social-determinants-of-health/demographic-change-and-healthy-ageing/social-isolation-and-loneliness: WHO; 2023.

[19] HM Government. A connected society. A strategy for tackling loneliness – laying the foundations for change Accessed from: https://assets.publishing.service.gov.uk/government/uploads/system/uploads/attachment_data/file/936725/6.4882_DCMS_Loneliness_Strategy_web_Update_V2.pdf: Crown; 2018.

[20] Department for Digital C, Media and Sport,. Tackling Loneliness annual report February 2022: the third year. Accessed from: https://www.gov.uk/government/publications/loneliness-annual-report-the-third-year/tackling-loneliness-annual-report-february-2022-the-third-year: Crown; 2022.

[21] Department for Digital C, Media and Sport,. Tackling Loneliness annual report March 2023: the fourth year. Accessed from: https://www.gov.uk/government/publications/loneliness-annual-report-the-fourth-year/tackling-loneliness-annual-report-march-2023-the-fourth-year#annex-a-government-action: Crown; 2023.

[22] Cattan M, Hogg E, Hardill I (2011). Improving quality of life in ageing populations: what can volunteering do?. Maturitas.

[23] Mountain G, Gossage-Worrall R, Cattan M, Bowling A. Only available to a selected few? Is it feasible to rely on a volunteer workforce for complex intervention delivery? Health & Social Care in the Community. 2017;25(1):177 – 84. 10.1111/hsc.12285 26445894

[24] Balki E, Hayes N, Holland C (2022). Effectiveness of Technology interventions in addressing social isolation, connectedness, and loneliness in older adults: systematic umbrella review. JMIR Aging.

[25] World Health Organisation. Evidence and gap map on digital Interventions for reducing social isolation and loneliness in older adults. Accessed from: https://www.who.int/initiatives/decade-of-healthy-ageing/evidence-gap-map/sil-digital: World Health Organisation; 2022.

[26] Barbosa Neves B, Waycott J, Malta S (2018). Old and afraid of new communication technologies? Reconceptualising and contesting the ‘age-based digital divide’. J Sociol.

[27] Waycott J, Vetere F, Pedell S, Morgans A, Ozanne E, Kulik L. Not For Me: Older Adults Choosing Not to Participate in a Social Isolation Intervention. Proceedings of the 2016 CHI Conference on Human Factors in Computing Systems; San Jose, California, USA: Association for Computing Machinery; 2016. p. 745–57.

[28] Barbosa Neves B, Waycott J, Maddox A (2021). When technologies are not enough: the challenges of Digital interventions to address loneliness in later life. Sociol Res Online.

[29] Department for Digital C, Media and Sport, Loneliness Annual Report. January 2020. Accessed from: https://www.gov.uk/government/publications/loneliness-annual-report-the-first-year/loneliness-annual-report-january-2020--2#ministerial-foreword-from-the-secretary-of-state-for-digital-culture-media-and-sport-and-the-minister-for-civil-society: Crown; 2020.

[30] Caprioli T, Giebel C, Reilly S, Tetlow H, Limbert S, Lloyd-Williams M (2023). Social support services for Dementia during the COVID-19 pandemic: a longitudinal survey exploring service adaptations in the United Kingdom. Health Expect.

[31] Naughton-Doe R, Wigfield A, Martin C (2023). Lessons from a voluntary sector organisation working to address loneliness and isolation among older people during the COVID-19 pandemic. Voluntary Sect Rev.

[32] Berkowsky RW, Rikard RV, Cotten SR. Signing off: Predicting discontinued ICT usage among older adults in assisted and Independent living. Springer International Publishing; 2015. pp. 389–98.

[33] Age UK, Briefing (2023). Poverty in later life.

[34] British Red Cross. A new Call to Action: Tackling Loneliness & Building Community. Accessed from: https://www.redcross.org.uk/loneliness-call-to-action: British Red Cross; 2023.

[35] Barnett J, Gillison F, Ellis D, Grey E, Baber F, Corbett E. Older adults and the pandemic: Tackling loneliness through technology. Available from: https://www.wcpp.org.uk/publication/older-adults-and-the-pandemic-tackling-loneliness-through-technology/: Wales Centre for Public Policy; 2022.

[36] Barnett J, Grey E, Baber F, Corbett E, Ellis D, Gillison F. Interview transcripts for the use of technology to address loneliness and social isolation among older adults: the role of social care providers. University of Bath Research Data Archive. 2023. 10.15125/BATH-01346. 10.1186/s12889-023-17386-w PMC10770975 38184519

[37] Braun V, Clarke V (2022). Conceptual and design thinking for thematic analysis. Qualitative Psychol.

[38] Ritchie J, Lewis J, McNaughton Nicholls C, Ormston R. Qualitative research practice: a guide for social science students and researchers. Second edition. ed. Los Angeles: SAGE; 2014.

[39] Broady T, Chan A, Caputi P (2010). Comparison of older and younger adults’ attitudes towards and abilities with computers: implications for training and learning. Br J Edu Technol.

[40] Centre for Ageing Better. The digital age: new approaches to supporting people in later life get online. Accessed from: https://ageing-better.org.uk/resources/digital-age2018.

[41] Harris MT, Blocker KA, Rogers WA. Older adults and Smart Technology: facilitators and barriers to Use. Front Comput Sci. 2022;4.

[42] Macedo IM (2017). Predicting the acceptance and use of information and communication technology by older adults: an empirical examination of the revised UTAUT2. Comput Hum Behav.

[43] Magsamen-Conrad K, Dowd J, Abuljadail M, Alsulaiman S, Shareefi A (2015). Life-span differences in the uses and gratifications of tablets: implications for older adults. Comput Hum Behav.

[44] Chen YR, Schulz PJ (2016). The Effect of Information Communication Technology Interventions on reducing social isolation in the Elderly: a systematic review. J Med Internet Res.

[45] Geerts N, Schirmer W, Vercruyssen A, Glorieux I. Exploring older adults’ ICT support: a mismatch between needs and provision. New Media & Society. 2023:14614448231166356.

[46] Thach KS, Lederman R, Waycott J. Adoption of videoconferencing for social connectedness among older adults: a systematic review. 2021.

[47] Zhao W, Kelly RM, Rogerson MJ, Waycott J. Older Adults Using Technology for Meaningful Activities During COVID-19: An Analysis Through the Lens of Self-Determination Theory. Proceedings of the 2023 CHI Conference on Human Factors in Computing Systems; Hamburg, Germany: Association for Computing Machinery; 2023. p. Article 332.

[48] Waycott J, Vines J, Neves BB, Vetere F (2019). Designing technologies with older adults: ethical tensions and opportunities. Ageing and Digital Technology: Designing and evaluating Emerging technologies for older adults.

[49] Blok M, van Ingen E, de Boer AH, Slootman M (2020). The use of information and communication technologies by older people with cognitive impairments: from barriers to benefits. Comput Hum Behav.

[50] Peek ST, Luijkx KG, Rijnaard MD, Nieboer ME, Van Der Voort CS, Aarts S (2016). Older adults’ reasons for using technology while aging in place. Gerontology.

[51] Tang X, Sun Y, Zhang B, Liu Z, LC R, Lu Z et al. “I Never Imagined Grandma Could Do So Well with Technology”: Evolving Roles of Younger Family Members in Older Adults’ Technology Learning and Use. Proc ACM Hum-Comput Interact. 2022;6(CSCW2):Article 478.

[52] Berridge C, Wetle TF (2020). Why older adults and their children disagree about In-Home Surveillance Technology, Sensors, and Tracking. Gerontologist.

[53] Thach KS, Lederman R, Waycott J. Personalizing Virtual Reality Experiences for Residents in Aged Care Homes: Lessons from a Case Study. Accessed from: https://aisel.aisnet.org/ecis2023_rp/331: ECIS 2023 Research Papers; 2023.

[54] Oliver K, Lorenc T, Tinkler J (2019). Evaluating unintended consequences: new insights into solving practical, ethical and political challenges of evaluation. Evaluation.

[55] Zamir S, Hennessy CH, Taylor AH, Jones RB (2018). Video-calls to reduce loneliness and social isolation within care environments for older people: an implementation study using collaborative action research. BMC Geriatr.

[56] Miller E, Wilding R, Baker S, Caldwell GA, Neves BB, Waycott J. Transforming aged care with virtual reality: how organisational culture impacts technology adoption and sustained uptake. Australas J Ageing. 2023. 10.1111/ajag.13248 37803886

[57] Budrikis A, Parry C, Adams C, Gringart E, Sim M, McAullay D (2023). Enabling social care services for older adults during periods of long-term social isolation: service provider perspectives. Australas J Ageing.

[58] Zamir S, Allman F, Hennessy CH, Taylor AH, Jones RB (2021). Aesthetically Designing Video-Call Technology with Care Home residents: a Focus Group Study. Front Psychol.

[59] Cattan M, Kime N, Bagnall A. Low-level support for socially isolated older people. An evaluation of telephone befriending Help the Aged; 2008. 10.1111/j.1365-2524.2010.00967.x 21114564

[60] Communications and Digital Committee. Digital exclusion. HL paper 219. Accessed from: https://publications.parliament.uk/pa/ld5803/ldselect/ldcomm/219/21902.htm: UK Parliament; 2023.

[61] Office for National Statistics. Measuring loneliness: guidance for use of the national indicators on surveys. Accessed from: https://www.ons.gov.uk/peoplepopulationandcommunity/wellbeing/methodologies/measuringlonelinessguidanceforuseofthenationalindicatorsonsurveys:ONS.gov.uk; 2018.

[62] McClelland H. The state of loneliness 2023: ONS data on loneliness in Britain June 2023. Campaign to End Loneliness; 2023.

[63] Patulny R, Bower M (2022). Beware the loneliness gap? Examining emerging inequalities and long-term risks of loneliness and isolation emerging from COVID-19. Australian J Social Issues.

[64] Rutter H, Savona N, Glonti K, Bibby J, Cummins S, Finegood DT (2017). The need for a complex systems model of evidence for public health. The Lancet.

[65] Fakoya OA, McCorry NK, Donnelly M (2020). Loneliness and social isolation interventions for older adults: a scoping review of reviews. BMC Public Health.

[66] Emerson E, Fortune N, Llewellyn G, Stancliffe R (2021). Loneliness, social support, social isolation and wellbeing among working age adults with and without disability: cross-sectional study. Disabil Health J.

[67] Niedzwiedz CL, Richardson EA, Tunstall H, Shortt NK, Mitchell RJ, Pearce JR (2016). The relationship between wealth and loneliness among older people across Europe: is social participation protective?. Prev Med.

