# Supplementary material for: The use of technology to address loneliness and social isolation among older adults: the role of social care providers
Source: BMC Public Health. 2024 Jan 6;24:108. doi: 10.1186/s12889-023-17386-w (PMC10770975; doi:10.1186/s12889-023-17386-w)
Supplement: Supplementary file 1 — Supplementary Material 1 [file 12889_2023_17386_MOESM1_ESM.pdf]

English

Default Question Block

.

Use of technology to tackle loneliness among older people during the coronavirus pandemic.

The purpose of this survey is to understand the ways in which technology might have been used in Wales to make social connections and seek to address loneliness and social isolation among community dwelling older people during the COVID-19 pandemic (i.e., from the first lock down in March 2020 to the present).

We would like to ask if, how, when and why your organisation has used technology with older adults (65+ years) and what impact this may have had on loneliness and social isolation. We are interested in uses of technology (such as phones and digital communication devices) that have helped to address loneliness and social isolation, even if they were not designed or intended specifically for this purpose. Before completing this survey, please make sure you have read the participant information sheet and privacy notice - you can download these by clicking the links below.

PARTICIPANT INFORMATION SHEET
TAFLEN WYBODAETH I GYFRANOGWYR

PRIVACY NOTICE
HYSBYSIAD PREIFATRWYDD

In this survey we use the term ‘older adult service users’ to mean any community dwelling older adults (i.e. not living in residential care or nursing homes) receiving care or support that you commission or that your organisation provides. This is a national survey, and we acknowledge that ‘older adult service users’ covers a huge range of ages and abilities/needs – when answering these questions, please think about the older adults that you provide services for.

..

Your organisation and your role

First, we would like to ask a few questions about your organisation and your role.

Q1. Which of the following best describes your role?

- ◯ I am an adult social care commissioner.
- ◯ I work for an organisation that provides adult social care.
- ◯ I work/volunteer for a community/voluntary sector organisation that provides support for older adults.
- ◯ Other (please specify)

Q2. Please briefly describe the population of older adults your organisation supports (e.g., "low income, varied education levels, many with mobility problems").

Q3. Which Local Authority is your organisation based in? (mark all that apply)

- ☐ Blaenau Gwent
- ☐ Bridgend
- ☐ Caerphilly
- ☐ Cardiff
- ☐ Carmarthenshire
- ☐ Ceredigion
- ☐ Conwy
- ☐ Denbighshire
- ☐ Flintshire
- ☐ Gwynedd
- ☐ Isle of Anglesey
- ☐ Merthyr Tydfil
- ☐ Monmouthshire
- ☐ Neath Port Talbot
- ☐ Newport

- ☐ Pembrokeshire
- ☐ Powys
- ☐ Rhondda Cynon Taf
- ☐ Swansea
- ☐ Torfaen
- ☐ Vale of Glamorgan
- ☐ Wrexham

Q4. Please state your job title in your organisation.

Q5. **In your role**, do you have direct contact (whether online, by phone or videocall, or face to face) with older adult service users?

- ○ No
- ○ Yes

Q6. **In your role,** during the pandemic, to what extent has preventing or reducing older adult service users' loneliness and social isolation been a priority? (Mark the box that best represents your view, with **1 = Not a priority at all,** and **7 = A primary priority**)

1 (Not a priority at all)	2	3	4	5	6	7 (A primary priority)
○	○	○	○	○	○	○

Q7. **In your organisation,** during the pandemic, to what extent has preventing or reducing older adults' loneliness and social isolation been a priority? (Mark the box that best represents your view, with **1 = Not a priority at all**, and **7 = A primary priority**)

1 (Not a priority at all)	2	3	4	5	6	7 (A primary priority)
○	○	○	○	○	○	○

.. **Technology in your organisation**

In order to help us understand how organisations may have used or encouraged use of technology with older adult service users, we first want to ask about what devices and

which software have been used to communicate between colleagues in your organisation or with other organisations throughout the pandemic.

Q8. In your organisation, how much are the following **devices and software** used to communicate *between colleagues in your organisation or with other organisations* (mark all that apply)

	Never	Very rarely	Rarely	Occasionally	Frequently	Ve[illegible] frequ[illegible]
Desktop computer	○	○	○	○	○	○
Laptop computer	○	○	○	○	○	○
Tablet/iPad	○	○	○	○	○	○
Landline telephone	○	○	○	○	○	○
Mobile phone (not smart phone)	○	○	○	○	○	○
Smart phone	○	○	○	○	○	○
Email (e.g., Microsoft Outlook, Gmail)	○	○	○	○	○	○
Video conferencing software (e.g., Microsoft Teams, Zoom, Skype, Facetime, WhatsApp call)	○	○	○	○	○	○
Text messaging to individuals or specific groups (including WhatsApp, Facebook direct messages etc.)	○	○	○	○	○	○
Social media posting (e.g., Facebook, Twitter, Instagram)	○	○	○	○	○	○
Other (please specify) [ ]	○	○	○	○	○	○

.. **Using technology with older adults**

We would now like to ask for your views about how – if at all – your organisation uses technology to communicate with older adult service users. This part of the survey is focused on communication in general, and is not focused on addressing loneliness and social isolation in particular.

Q9. In your organisation, how often **do you or your colleagues** use any of the following devices and software to communicate *with older adult service users*?

	Never	Very rarely	Rarely	Occasionally	Frequently	Very frequently
Desktop computer	○	○	○	○	○	○
Laptop computer	○	○	○	○	○	○
Tablet/iPad	○	○	○	○	○	○
Landline telephone	○	○	○	○	○	○
Mobile phone	○	○	○	○	○	○
Smart phone	○	○	○	○	○	○
Email (e.g., Microsoft Outlook, Gmail)	○	○	○	○	○	○
Video conferencing software (e.g., Microsoft Teams, Zoom, Skype, Facetime, WhatsApp call)	○	○	○	○	○	○
Text messaging to individuals or specific groups (including WhatsApp, Facebook direct messages etc.)	○	○	○	○	○	○
Social media posting (e.g., Facebook, Twitter, Instagram, Next Door)	○	○	○	○	○	○
Other (please specify) ________	○	○	○	○	○	○

Q10. In your experience, approximately what proportion of older adult service users use the following **devices and software** to communicate with other people (e.g., friends, family, professionals)? We realise you will probably not have an exact knowledge of this – we are interested in your general impression. (**1 = None of our older adult service users** and **7 = All of our older adult service users**)

	1 (None)	2	3	4	5	6	7 (All)	Don't know
Desktop computer	○	○	○	○	○	○	○	○
Laptop computer	○	○	○	○	○	○	○	○
Tablet/iPad	○	○	○	○	○	○	○	○
Smart phone	○	○	○	○	○	○	○	○
Mobile phone	○	○	○	○	○	○	○	○
Landline telephone	○	○	○	○	○	○	○	○
SmartHome devices (Amazon Echo, Google Home)	○	○	○	○	○	○	○	○

	1 (None)	2	3	4	5	6	7 (All)	Don't know
Email (e.g., Microsoft Outlook, Gmail)	○	○	○	○	○	○	○	○
Video conferencing software (e.g., Microsoft Teams, Zoom, Skype, Facetime, WhatsApp call)	○	○	○	○	○	○	○	○
Text messaging to individuals or specific groups (including WhatsApp, Facebook direct messages etc.)	○	○	○	○	○	○	○	○
Social media posting (e.g., Facebook, Twitter, Instagram, Next Door)	○	○	○	○	○	○	○	○
Other (please specify) ____	○	○	○	○	○	○	○	○

.. Loneliness and social isolation among older adults.

Q11. Does your organisation assess/measure loneliness and social isolation in older adult service users?

- ○ No
- ○ Yes

Q11.2. Please tell us how you do this?

____

Q12. To what extent do you believe the **radio** has a role in preventing or reducing social isolation in your older adult service users?

- ○ A great deal
- ○ Considerably
- ○ Moderately
- ○ Slightly
- ○ Not at all

Q13. To what extent do you believe the **TV** has a role in preventing or reducing social isolation in your older adult service users?

- A great deal
- Considerably
- Moderately
- Slightly
- Not at all

.. We are now interested in whether your organisation has used devices and software to address loneliness and social isolation among older adult service users.

Q14. Has your organisation sought to *prevent or reduce older service users' experiences of loneliness and social isolation* by encouraging or enabling them to link with people outside your organisation (e.g. support groups, friends, family, other service users)?

- No
- Yes

Q14.2. Please can you tell us a little more detail to explain your answer.

Q15. For each of the options below, please indicate how often they have been used **by your organisation** to communicate with older adult service users to *prevent or reduce experiences of loneliness and social isolation.*

	Never	Very rarely	Rarely	Occasionally	Frequently	Very frequently
Desktop computer						
Laptop computer						
Tablet/iPad						
Landline telephone						
Mobile phone						
Smart phone						
Email (e.g., Microsoft Outlook, Gmail)						

	Never	Very rarely	Rarely	Occasionally	Frequently	Very frequently
Video conferencing software (e.g., Microsoft Teams, Zoom, Skype, Facetime, WhatsApp call)	○	○	○	○	○	○
Text messaging to individuals or specific groups (including WhatsApp, Facebook direct messages etc.)	○	○	○	○	○	○
Social media posting (e.g., Facebook, Twitter, Instagram, Next Door)	○	○	○	○	○	○
Other (please specify)	○	○	○	○	○	○

.. In the following questions we would like to know more about the use of technology to prevent or reduce loneliness and social isolation.

Q16. When did your organisation start using technology with older adult service users for the purpose of helping prevent or reduce loneliness and social isolation?

- ○ Before the first lockdown of the COVID-19 pandemic in March 2020
- ○ Since the first lockdown of the COVID-19 pandemic in March 2020

Q17. We are interested in how technology has been applied to prevent or reduce loneliness and social isolation in older adult service users since the first lockdown of the COVID-19 pandemic. To the best of your knowledge, how much has technology been used for the following purposes in your organisation?

	A great deal	Considerably	Moderately	Slightly	Not at all
To share information with older adult service users about available support (e.g. other services)	○	○	○	○	○
To engage in one-to-one conversations with older adult service users	○	○	○	○	○
To enable older adult service users to maintain links with friends and family	○	○	○	○	○

	A great deal	Considerably	Moderately	Slightly	Not at all
To enable older adult service users to link up with individuals they did not know prior to the pandemic	○	○	○	○	○
To enable older adult service users to link up with groups they belonged to that previously met face to face	○	○	○	○	○
To enable older adult service users to link up with new groups set up since the start of the pandemic	○	○	○	○	○

Q18. Were there any barriers that made using technology difficult for your older adult service users during the pandemic? Please indicate the extent to which you think each of the following were an issue for your older adult service users.

	A great deal	Considerably	Moderately	Slightly	Not at all
Hardware/software problems (e.g., lack of devices, software incompatible with devices)	○	○	○	○	○
Lack of internet access or data	○	○	○	○	○
Limited technological skills	○	○	○	○	○
Lack of confidence in using technology	○	○	○	○	○
Lack of interest in/desire to use technology	○	○	○	○	○
Other (Please specify) [text box]	○	○	○	○	○

Q19. Have your older adult service users **required** any support *to use technology to connect with others* during the pandemic?

	Support was required
Provision of devices or software	☐
Technical support to set up devices/software	☐

	Support was required
One to one help and support	☐
Encouragement	☐
Financial support (e.g. to pay for internet, data or phone contract)	☐
Other (please specify)	☐

Q20. Have your older adult service users **been provided with** any support *to use technology to connect with others* during the pandemic? (Please mark the columns to show from whom support has been provided)

	Support provided by our organisation	Support provided by another organisation	Support provided by family/friends	Support required but not provided
Provision of devices or software	☐	☐	☐	☐
Technical support to set up devices/software	☐	☐	☐	☐
One to one help and support	☐	☐	☐	☐
Encouragement	☐	☐	☐	☐
Financial support (e.g. to pay for internet, data or phone contract)	☐	☐	☐	☐
Other (please specify)	☐	☐	☐	☐

Q21. As far as you know, how many of your older adult service users have started to use devices or applications that they didn't use before the pandemic to connect with others?

- ○ Many have
- ○ Some have
- ○ A few have
- ○ None have
- ○ I have no idea

Q22. As far as you know, how many of your older adult service users have started to use devices or applications to connect with others in different ways than they did before the pandemic?

- Many have
- Some have
- A few have
- None have
- I have no idea

Q23. In your experience, how appropriate do you feel it is to use technology to communicate with older adult service users?

- Extremely appropriate
- Appropriate
- Neither appropriate nor inappropriate
- Inappropriate
- Extremely inappropriate

Q23.2. Please explain your answer.

Q24. In your experience, how confident have you felt using technology to communicate with older adult service users?

- Extremely confident
- Confident
- Neither confident or unconfident
- Unconfident
- Extremely unconfident

Q24.2. Please explain your answer.

Q25. Are there groups of older adult service users for whom the use of technology to connect with others had a particularly **positive impact**? If so, please provide brief

details.

Q26. Are there groups of older adult service users for whom the use of technology to connect with others was **not appropriate** or had a **negative impact**? If so, please provide brief details.

Q27. Has your organisation formally or informally evaluated the impact of older adult service users using technology for preventing or reducing loneliness and social isolation? (Mark one box only)

- ○ No evaluation and no feedback gathered.
- ○ Informal feedback gathered (e.g., listening to anecdotal comments)
- ○ Formally evaluated (i.e., information purposely gathered to understand effect of technology use)
- ○ Formal evaluation and informal feedback gathered.

Q28. What did you find out from the information that you gathered? Please tell us some details below

Q29. If there is a report of your evaluation, we would love to see it – if that is possible, please send this to yourviews@bath.ac.uk

Q30. Do you think the use of technology was beneficial in helping your organisation to prevent or reduce loneliness and social isolation for older adult service users?

- ○ Extremely beneficial
- ○ Very beneficial
- ○ Moderately beneficial
- ○ Slightly beneficial
- ○ Not at all beneficial

Q31.2. Please explain your answer.

.. Face-to-face support and use of technology in the future

Q32. We recognise that during the pandemic it has been extremely difficult to deliver face-to-face support. Before the pandemic what was the balance of face to face and remote support your organisation provided for older adult service users?

- Fully face-to-face
- Mostly face-to-face
- Equal balance of face-to-face and remote
- Mostly remote
- Fully remote

Q33. Looking ahead to the coming months, what do you anticipate the balance of face to face and remote support your organisation provides for older adult service users will be?

- Fully face-to-face
- Mostly face-to-face
- Equal balance of face-to-face and remote
- Mostly remote
- Fully remote
- Not applicable

Q34. Of the older adult service users you supported before the pandemic, roughly what proportion did you keep in contact with using technology throughout the pandemic?

0%	20%	40%	60%	80%	100%
O	O	O	O	O	O

Q35. Of those you did not keep in contact with using technology, please tell us the reasons for this.

Q36. Are there any technologies you have not used thus far that you would be interested in adopting *in your organisation* to help prevent and reduce loneliness and social isolation among older adults?

- No

- Yes

Q37. Which kinds of technologies would you like to use?

Q38. Do you think technology should be used more to help prevent or reduce loneliness and isolation among your older adult service users?

- No
- Yes

Q39. Please explain your answer.

Q40. Are you aware of any organisations in your local authority (other than your own) currently using devices or software to prevent or reduce loneliness and social isolation among older adults?

- No
- Yes

Q41. Please can you tell us the names of those organisations and what they are doing.

Q42. Are there any technologies you think should be considered/tried to help reduce loneliness and social isolation among older adults?

- No
- Yes

Q43. Please provide details.

Q44. What is the best example of preventing or reducing loneliness and social isolation using technology that you know of? Please provide brief details.

Q46. Do you think the use of technology could be beneficial in helping your organisation to prevent or reduce loneliness and social isolation for older adult service users?

- Extremely beneficial
- Very beneficial
- Moderately beneficial
- Slightly beneficial
- Not at all beneficial

Q46.2. Please explain your answer.

Q47. Do you think technology should be used more to help prevent or reduce loneliness and isolation among your older adult service users?

- No
- Yes

Q47.2. Please explain your answer.

Q48. In your experience, how appropriate do you feel it is to use technology to communicate with older adult service users?

- Extremely appropriate
- Appropriate
- Neither appropriate nor inappropriate
- Inappropriate
- Extremely inappropriate

Q49. Please explain your answer.

Q50. Does your organisation have plans to start using technology to prevent or reduce social isolation and loneliness for older adult service users?

- No
- Yes
- Don't know

Q51. Are you aware of any other organisations in your local authority currently using devices or software to prevent or reduce loneliness and social isolation among older adults?

- No
- Yes

Q52. Please can you tell us the names of those organisations and what they are doing

Q53. Are there any technologies that you think should be considered/tried to help reduce loneliness and social isolation among older adults? If yes, please provide details.

Q54. What is the best example of preventing or reducing loneliness and social isolation using technology that you know of? Please provide brief details.

..

Thank you so much for completing the survey so far. We now have a few questions about you.

Your responses to these questions will help us understand if/how people's characteristics impact on their use of technology in work.

Q55. What gender best describes how you see yourself?

- ○ Male
- ○ Female
- ○ Non-binary / third gender
- ○ Prefer not to say

Q51. What is your age?

- ○ 18-24
- ○ 25-34
- ○ 35-44
- ○ 45-54
- ○ 55-64
- ○ 64+

Q52. How long have you worked in or with the care, voluntary or community sector?

- ○ Less than 2 years
- ○ 2-5 years
- ○ 5-10 years
- ○ 10-15 years
- ○ 15-20 years
- ○ More than 20 years

Q53. Finally, as mentioned in the information sheet, we are very keen to interview some survey respondents to find out a bit more about your experiences and understanding of technology, loneliness and social isolation among older adults in Wales. If you would be willing to take part in an interview via telephone or video call (which would last about 45 minutes) please leave your contact details below.

Name __________

Email address __________

Telephone number __________

Q54. We are also keen to include some ‘case studies’ of promising examples of using technology to prevent or reduce loneliness and social isolation among older adults. These would be short descriptions of what has been done and what impact it has had.

If you would be happy for us to contact you about a case study, please leave your contact details below.

Name

Email address

Telephone number

Name of organisation

Job title

Brief description of case study

Q55. We would be happy to let you know the results of this survey if you are interested. If you would like to receive a copy, please leave your name and email address below.

Name

Email address

Q56. If you would like to be entered into the prize draw for a chance of winning one of three vouchers (£50, £30 or £20), please leave your name and email address below.

Name

Email address

.. Thank you again, we really appreciate the time you have taken to complete this survey.

Powered by Qualtrics
