# Supplementary material for: The use of technology to address loneliness and social isolation among older adults: the role of social care providers
Source: BMC Public Health. 2024 Jan 6;24:108. doi: 10.1186/s12889-023-17386-w (PMC10770975; doi:10.1186/s12889-023-17386-w)
Supplement: Supplementary file 2 — Supplementary Material 2 [file 12889_2023_17386_MOESM2_ESM.docx]

# Interview schedule – service providers

## Interview schedule

[Prior to the interview, the researcher will have reviewed the interviewee’s survey responses, particularly their job role, whether or not they have used tech with older adults and if they evaluated this, and their perceptions of barriers/positive impacts]

Thank you for taking part in this project. As you know, we are interested in your experiences and understanding of technology, loneliness and social isolation among older people in Wales. In this call, I’ll ask you some questions about this. There are no right or wrong answers, we are just interested in understanding your experiences. I’ll record what is said in this call so that it can be typed up later, but any information that could identify you, such as names of people or places, will be removed. If at any time during the call you would like to stop, just let me know, and you do not have to answer any questions that you do not feel comfortable with.

Before we start the questions, I just need to run through the consent statements that were attached to the information sheet. I’ll read each statement out and if you could just state that you agree to each, or ask me to clarify anything.

→ refer to consent form

[Begin recording]

*Background/scene setting*

1. To start, please could you give a brief overview of your role and of the organisation that you work for.
2. Could you tell me about the work that your organisation does (or the organisations you commission do) with older people? *(Prompt – (if applicable) how does this differ, if at all, from the work you do with other populations?)*
   - How much focus is there on preventing loneliness and social isolation? (*Prompts – which groups most affected)*

*Role of digital in preventing/mitigating loneliness*

1. If you think back to the early days of COVID-19, what sorts of conversations were had in your organisation about how digital technology could/should be used to prevent or mitigate loneliness or social isolation? (P*rompts – how different was this from what you were doing pre-covid? Did you have a choice in what or how technology would be used, or was this mandated?)*
   - What did you and your colleagues think were the pros and cons of using technology to this end? *(Prompts – what were these views based on – what others said, personal experience?)*
   - Why did you decide to use/not to use tech? [refer to survey responses on whether or not they used tech – Q14-17] *(Prompts - What did you consider, was access to hardware and software discussed, was data security discussed, what evidence did they base their decision on?)*
   - Have you tried any tech previously that hasn’t worked?
   - Why did it not work for them (e.g. did it not meet their users’ needs, was there a lack of long term support etc)?
2. [If they DID use tech] Can you tell me some more details of the main ways digital technology was used in your organisation to address loneliness and social isolation during COVID-19? *(Prompts - what was done, why, using what platforms, with whom, how often)*.
   - Overall, did you achieve your goal; do you consider this was a success? *(Prompts: what does success mean?)*
   - Was it more successful at reducing loneliness and social isolation for some groups compared to others? *(prompt: who did it work well/less well for; why do you think this was?)* [Q25 &26]
   - Of those service users who did engage with the technology, what do you think was their motivation to do so and to keep engaging?
   - If there were any service users who tried using technology but then stopped, do you know why they stopped? What do you think could be done, if anything, to help these people continue to engage with the service through technology?
   - If your service users encountered any technical problems, what support was provided (if any)? [Q18-20]
   - One issue we know to be a barrier for some older adults is a fear of using technology, either due to not wanting to break the devices or being afraid of internet scams. Was this an issue for the older adults you work with and, if so, what (if anything) was done to help overcome these fears?
   - What effect did using technology in this way have on you and your colleagues and the work you do?
3. Were there any **unexpected positive effects** of using/not using digital tech to address L&SI? Were there any **unexpected negative effects** of using/not using digital tech to address L&SI? *(Prompts – details?)*
4. In your survey responses, you said that you anticipate the balance of face-to-face and remote support your organisation provides in the coming months would be [Q33] – why have you decided to go with/not go with a blend of both face-to-face and remote? *(prompt: what would be needed to make a using both face-to-face and remote delivery successful for your service users and organisation, or do you feel this isn’t appropriate?)*
5. [If they will use tech in future] Do you plan to do any formal evaluation of loneliness and social isolation of your service users and/or the [technology they plan to use]?
   - [If yes] How will you do this? Is there any support that would help you do this?
   - [If no] Why not? Is there any support that could help you do this?

**Thanks very much and close.**
